# Methamphetamine enhances HIV infection of human iPSC-derived microglia

**DOI:** 10.1515/nipt-2026-0003

**Published:** 2026-03-02

**Authors:** Xiao-Long Wang, Xu Wang, Yuan Rong, Wen-Zhe Ho

**Affiliations:** Department of Pathology and Laboratory Medicine, Temple University, Philadelphia, PA, USA; Department of Pathology and Laboratory Medicine, Temple University, Philadelphia, PA, USA; Department of Pathology and Laboratory Medicine, Temple University, Philadelphia, PA, USA; Department of Pathology and Laboratory Medicine, Temple University Medical School, Philadelphia, PA, USA

**Keywords:** methamphetamine, human immunodeficiency virus, interferon-stimulated genes, induced pluripotent stem cell lines (iPSCs), microglia

## Abstract

Methamphetamine (METH) is a potent psychostimulant that is commonly used by people infected with HIV. Clinically, METH use is implicated in HIV infection and neuroinflammation. In this study, we examined whether METH has direct effect on HIV infection of human microglia, the major target and reservoir cells for the virus in the brain. We observed that METH treatment of human iPSC-derived microglia (iMg) significantly enhanced HIV replication, as indicated by increased HIV gag expression, p24 protein levels, and reverse transcriptase activity. Mechanistically, METH suppressed the expression of interferons (IFNs), IFN stimulated gene (Viperin) and the CC chemokine (RANTES). In addition, METH upregulated the expression of the HIV entry coreceptors (CCR5 and CXCR4) in iMg. These findings suggest that METH use is a promoting factor for HIV infection of microglia. Because many individuals infected with HIV use METH, it is important to further investigate the interactions between METH use and HIV in target cells to better understand the mechanisms underlying HIV persistence in the brain and to develop effective strategies for viral eradication.

Methamphetamine (METH) abuse is prevalent among people living with HIV. It has been demonstrated that METH induces inflammatory cytokine production, impairs immune function, and promotes HIV infection, thereby contributing to the development of NeuroHIV [1]. Although METH use has been linked to increased HIV transmission and viral replication, its pathological effects on host cell susceptibility to HIV infection remain incompletely understood [2]. Previous studies have reported that METH enhances HIV infection in several cell types, including dendritic cells [3], macrophages [4, 5], monocytes [6], CD4+ T cells [7, 8], and neural progenitor cells [9]. However, whether METH facilitates HIV infection of human microglia – the principal target and reservoir cells for HIV in the brain – remains unclear.

As the resident immune cells of the brain, microglia play critical roles not only in brain immunity but also in synaptic plasticity and neurogenesis [4]. Studies of HIV infection in microglia have been limited by the restricted availability of primary human microglial cells. This challenge, together with the increasing emphasis on reducing the use of rodent animal models, underscores the need for renewable and well-validated human microglial models. Recently, induced pluripotent stem cell–derived microglia (iMg) have emerged as a valuable platform for modeling neurological diseases, providing a stable and physiologically relevant source of human microglia for investigating brain disease mechanisms [10]. Importantly, iMg has been shown to be highly susceptible to HIV infection and to more closely resemble authentic human microglia than transformed microglial cell lines [11, 12]. Therefore, in this study, we employed iMg to determine whether METH has a direct effect on HIV infection of human microglia and to elucidate the underlying mechanisms.

## METH facilitates HIV infection in iMg

**Figure 1:**
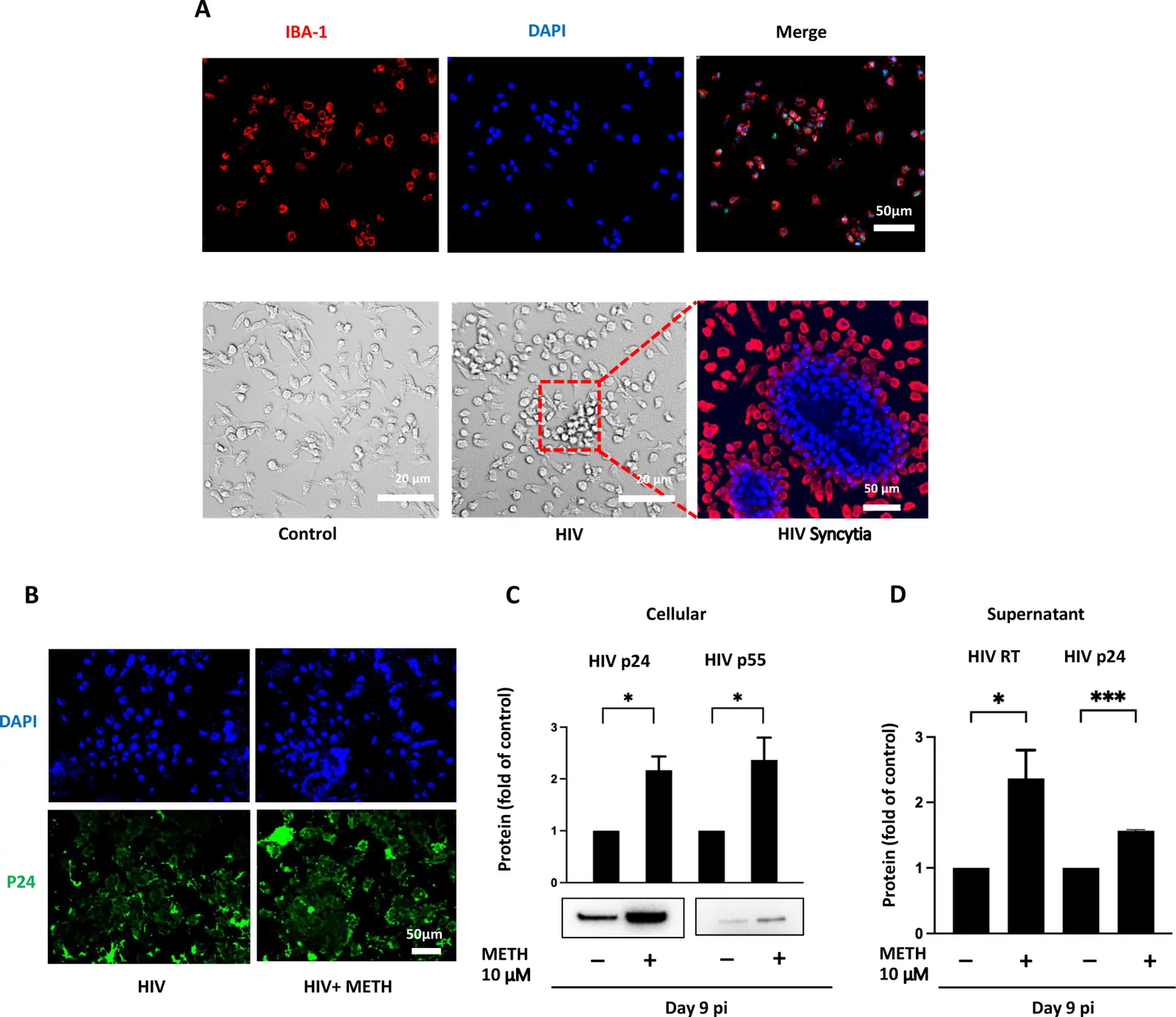
METH enhances HIV infection of iMg. (A) iMg were immunostained with antibodies targeting the microglial marker IBA-1. Cell nuclei were counterstained with Hoechst (DAPI). Scale bar: 50 μm. Representative confocal images obtained 9 days post-HIV infection (MOI of 0.018) demonstrate the formation of giant syncytia (indicated by red frames), consistent with HIV-induced cytopathic effects. (B) Immunofluorescence staining of HIV-infected and uninfected iMg fixed 9 days post-infection. Cells were stained for HIV Gag p24 antigen (green). Scale bar: 50 μm. (C) iMg were infected with HIV and treated with or without METH (10 μM). Intracellular HIV Gag protein expression was assessed by Western blot analysis. Cell lysates from iMg with and without METH treatment were collected and analyzed by Western blotting for HIV Gag p24, Pr55, and GAPDH. Data represents SD from three independent experiments performed in triplicate. (D) Reverse transcriptase (RT) activity assays (*p<0.05) and ELISA p24 levels (^***^p<0.001) detected in the supernatant at day 9pi. Data represent mean ± SD of triplicate cultures, expressed as fold change relative to control.

To assess the permissiveness of iMg to HIV infection, we first confirmed cellular phenotype via immunofluorescence microscopy using the microglia-specific marker IBA-1. Following inoculation with HIV strain Bal, characteristic syncytium formation was observed, confirming productive viral entry (Figure 1A). Immunofluorescence analysis of the HIV capsid protein p24 in infected iMg demonstrated that METH exposure augmented viral protein accumulation and fluorescence intensity without compromising cell survival (Figure 1B). As demonstrated in Figure 1C and D, METH treatment substantially elevated HIV p24 protein levels in iMg, with enhanced expression detected in both cellular homogenates (intracellular compartment) and cell culture media (extracellular compartment).

## METH modulates innate immune responses in HIV-infected iMg

**Figure 2:**
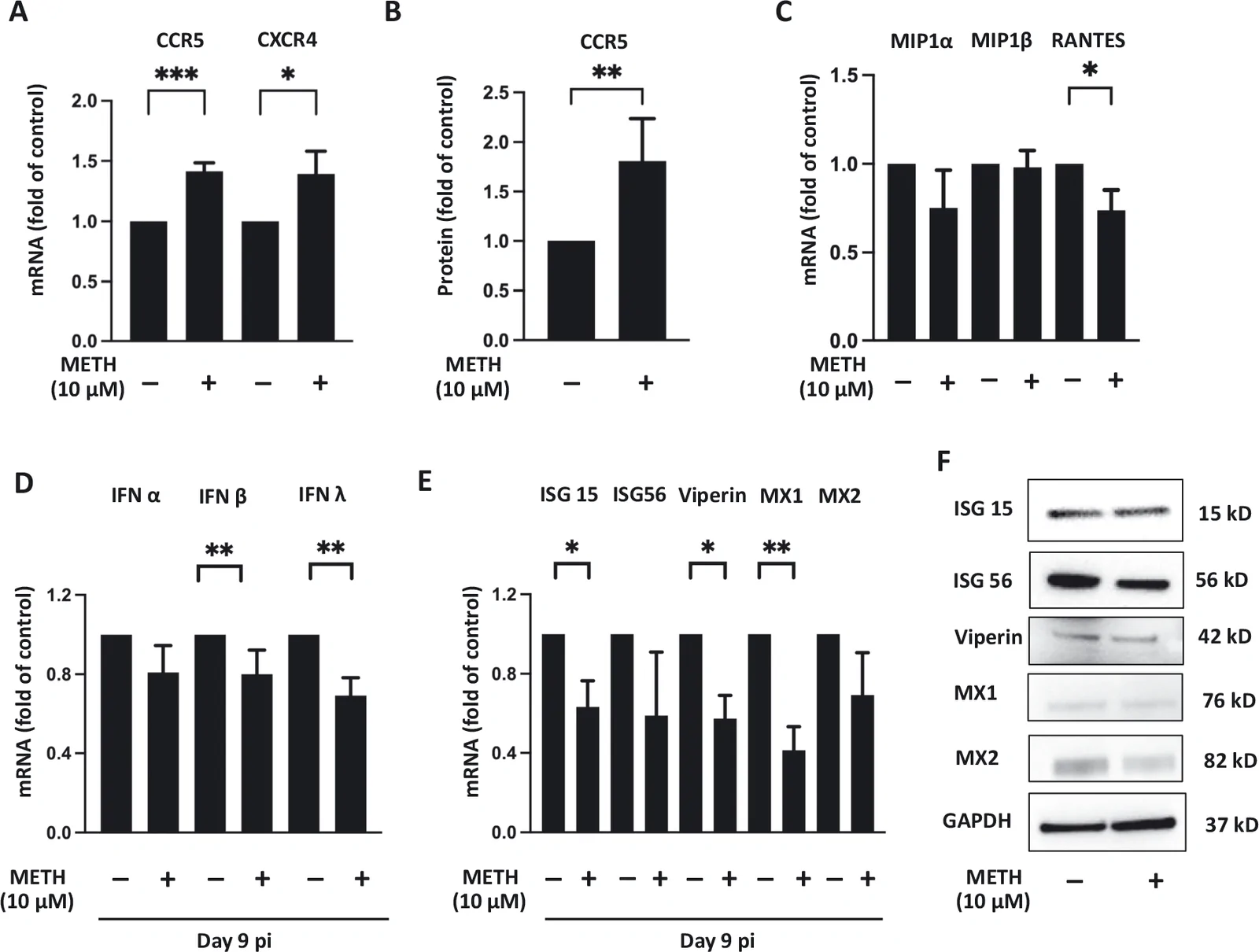
METH suppresses antiviral immune responses in HIV-infected iMg. (A) iMg were treated with 10 μM METH for 24 h. Total RNA was extracted and analyzed by real-time PCR to assess CXCR4 and CCR5 mRNA expression (*p<0.05, ^***^p<0.001). (B) Corresponding cell lysates were analyzed by Western blotting to evaluate CCR5 and GAPDH protein levels. Results demonstrate that METH treatment significantly upregulated CCR5 protein expression (^**^p<0.01). (C) Real-time PCR analysis of CC chemokines revealed that METH treatment decreased RANTES expression in uninfected iMg (*p<0.05). Data represents SD from three independent experiments. (D and E) RT-PCR and Western blot analyses were performed on day 9 pi to measure mRNA levels of interferon genes (IFN-β and IFN-λ) and interferon-stimulated genes (ISG15, Viperin, and Mx1). Significant changes are indicated (*p<0.05, ^**^p<0.01). (F) Western blot analysis of total protein lysates was performed to assess ISG expression levels between METH-treated and control groups, with representative blots presented.

To elucidate the molecular mechanisms underlying METH-enhanced HIV entry into microglia, we investigated viral entry receptor and chemokine expression profiles following our established infection protocol [11]. METH exposure resulted in substantially elevated p24 expression, concurrent with significant upregulation of the HIV co-receptors CCR5 (p<0.001) and CXCR4 (p<0.05) (Figure 2A). Corresponding CCR5 protein abundance was similarly increased (Figure 2B). Since CC chemokines (MIP-1α, MIP-1β, and RANTES) function as physiological ligands for the HIV co-receptor CCR5 and serve as competitive inhibitors of viral entry, we assessed whether METH modulates CC chemokine production in iMg. METH treatment induced substantial downregulation of RANTES mRNA expression (p<0.05) (Figure 2C), suggesting that diminished chemokine-mediated competition may facilitate enhanced viral entry. Collectively, these results indicate that METH potentiates HIV infection in iMg through coordinated alterations in co-receptor and chemokine expression that favor viral accessibility.

## METH suppresses IFNs and ISGs responses in HIV-infected iMg

To identify potential mediators contributing to METH-enhanced HIV replication, we evaluated the impact of METH treatment on IFN and IFN-stimulated gene (ISG) expression. METH exposure resulted in marked suppression of mRNA expression for IFN-β (p<0.01) and IFN-λ (p<0.01) (Figure 2D). Furthermore, METH significantly attenuated the expression of multiple antiviral ISGs (ISG-15, ISG56, Viperin, Mx1, and Mx2) at 9 days post-infection, with ISG15, Viperin, and Mx1 showing substantial downregulation (p<0.05) (Figure 2E). Corresponding protein levels of these ISGs were reduced following METH treatment, consistent with the transcriptional observations (Figure 2F).

Collectively, these findings establish that METH impairs the innate immune response in microglia, thereby compromising antiviral defense mechanisms and promoting HIV replication. METH exerts a dual modulatory effect by simultaneously enhancing viral entry and replication whilst suppressing type I/III IFN expression and critical antiviral ISGs, including ISG15, Viperin, and Mx1. Because these ISGs function as essential restriction factors that inhibit HIV replication at multiple stages of the viral life cycle, their downregulation consequently compromises microglia-mediated innate antiviral immunity in the brain. Concurrently, METH upregulates the HIV entry co-receptors CCR5 and CXCR4 while simultaneously downregulating RANTES, an endogenous antagonist of viral entry, thereby facilitating HIV infection. In aggregate, these integrated molecular and cellular alterations establish a permissive microenvironment for HIV replication within the central nervous system, elucidating the mechanisms underlying NeuroHIV progression in METH-consuming individuals. Despite METH’s potent addictive properties as a psychostimulant and documented association with high-risk behavioral practices, METH consumption remains highly prevalent among HIV-infected populations [1]. Clinically, METH exposure has been associated with systemic immune dysregulation and enhanced HIV replicative capacity [2]. This study definitively establishes that METH exacerbates both HIV infection and HIV-associated neurocognitive disorder (HAND). By elucidating the direct mechanistic effects of METH on HIV infection within microglia – the primary cellular targets and viral reservoirs in the brain – this work underscores the critical role of METH in accelerating NeuroHIV pathogenesis.

Notably, our utilization of iMg provides a physiologically relevant and experimentally tractable platform that faithfully recapitulates the cellular and molecular phenotypes characteristic of human brain tissue [11]. This model system circumvents the inherent limitations associated with primary iMg preparations and facilitates comprehensive mechanistic investigations whilst eliminating dependence on animal experimentation. In the context of heightened regulatory scrutiny regarding the reduction and refinement of animal-based research paradigms, our approach represents a contemporaneous, ethically principled alternative for interrogating HIV infection and replication within the central nervous system [13]. This work constitutes a mean-ingful advancement in NeuroHIV research, enabling granular dissection of viral-host molecular interactions in neural tissue and establishing a scalable experimental platform for future translational and therapeutic development initiatives.

## Supplementary Material

Supplement

**Supplementary Material:** This article contains supplementary material (https://doi.org/10.1515/nipt-2026-0003).
