# Supplementary material for: Methamphetamine enhances HIV infection of human iPSC-derived microglia
Source: NeuroImmune Pharm Ther. Author manuscript; Available in PMC 2026 Sep 5. (PMC13544641; doi:10.1515/nipt-2026-0003)
Supplement: Supplementary file 1 [file NIHMS2203097-supplement-Supplement.pdf]

Methods and Materials

1 Cells and Reagents

iPSCs were obtained from Human Immunology Core at the University of Pennsylvania (Philadelphia, PA, USA). The Core has the Institutional Review Board approval for blood collection from healthy donors. Whole blood samples were collected by the Children's Hospital of Philadelphia (CHOP). Noteworthy, these blood samples were screened for all normal viral bloodborne pathogens and certified to be pathogen free. All studies involving iPSCs were performed under approved protocols of the University of Pennsylvania and NIH. All iPSC lines were confirmed to have a normal karyotype and were confirmed free of mycoplasma, acholeplasma, and piroplasm with a detection limit of 10 CFU/mL by targeted PCR (Biological Industries). iPSCs were differentiated into CMPs according to the published protocol by the Human Pluripotent Stem Cell Core in CHOP. CMPs were plated at 5.6×10^4 cells/well in a 96-well CellBIND plate (Corning 3330). CMPs were differentiated in fresh iMg medium (RPMI 1640 medium, GE Healthcare Life Sciences, SH30027.01) with 10% FBS (HyClone, SH30071.03HI), recombinant human IL-34 (100 ng/mL, R&D Systems, 5265-IL-010), M-CSF-1 recombinant human protein (25 ng/mL, Thermo Fisher Scientific, PHC9504), and recombinant human TGF-β1 (50 ng/mL, PeproTech, 100-21). Half medium changes were performed every 2 days at the early stage of differentiation and fully changed after day 6. Eventually, microglia differentiation was completed and accessible for treatment/infection on day 10.

Rabbit antibodies against ISG15, ISG56, Viperin, Mx1, Mx2, and GAPDH were purchased from Cell Signaling Technology (Danvers, MA, USA). Mouse anti-HIV p24 antibody was purchased from Abcam (Abcam, Cambridge, UK). METH was purchased from Sigma Aldrich (St Louis, MO, USA). METH powder was dissolved in sterile endotoxin-free water (Hy-Pure, TM Cell culture grade water, GE Healthcare Life Science, Logan, UT, USA) and stored at 4 °C. Trichloroacetic acid (TCA) and acetone were purchased from Sigma-Aldrich.

2 HIV Infection and METH Treatment

HIV BaL strain, obtained from the NIH AIDS Reagent Program (Bethesda, MD), was used for all infection experiments. iPSC-derived microglia (iMg) were generated from iPSC lines procured

through the Human Immunology Core at the University of Pennsylvania (Philadelphia, PA, USA), which holds Institutional Review Board approval for blood collection from healthy donors. Source whole blood samples were collected at the Children's Hospital of Philadelphia (CHOP) and were pre-screened and certified pathogen-free for all standard viral bloodborne pathogens. iPSCs were differentiated into common myeloid progenitors (CMPs) in accordance with the established protocol of the Human Pluripotent Stem Cell Core at CHOP, and CMPs were subsequently seeded at 5.6×10^4 cells/well in 96-well CellBIND plates (Corning 3330) to undergo terminal differentiation into iMg. Prior to infection, iMg (approximately 1.7×10^5 cells total) were exposed to 10 μM methamphetamine (METH) for 24 hours, a pretreatment paradigm consistent with that applied to primary monocytes. Cells were subsequently challenged overnight with HIV BaL at a multiplicity of infection (MOI) of 0.018, calculated from a p24-normalized inoculum of 1.8 ng/10^6 cells: the total p24 delivered to 1.7×10^5 cells was 0.306 ng, which was converted to approximately 3,060 infectious units (IU) based on the standard conversion factor of 1 ng p24 $\approx 10^4$ IU, yielding an MOI of 0.018. METH exposure was thus maintained continuously across both the 24-hour pretreatment period and the subsequent overnight infection, ensuring that cells remained pharmacologically conditioned throughout the early phase of viral challenge. The cells were then washed three times with plain RPMI/DMEM to remove any unabsorbed virus and cultured in the presence of METH. Viral titers were determined by using ELISA assay and RTA assay. Readouts were performed on wells with the highest dilution displaying observable infection.

3 Western Blot Assay

For the Western blot analysis, samples from iMg were homogenized in the RIPA lysis buffer (Sigma Aldrich, MO, USA) supplemented with protease/phosphatase inhibitors (Sigma Aldrich, MO, USA). Along with this, the protein concentrations were examined by the bicinchoninic acid (BCA) assay (Chem-Cruz, Dallas, TX). After boiling with NuPAGE™ LDS Sample Buffer and NuPAGE™ Sample Reducing Agent (Thermo Scientific™, MA, USA), 20 µg of protein/ lane of each sample was subject to pre-cold 12% SDS-PAGE gel (Thermo Scientific™, MA, USA). IMg and culture supernatants were subjected to Western blot assay for investigating the viral proteins (p24 and Pr55), CC Chemokines, and ISGs expression. The blots were incubated with primary antibodies in 5% non-fat milk (Cell Signaling Technology, Inc. MA, USA) in PBS overnight at 4 °C, then washed with PBS containing 0.5% Tween 20 (Bio-Rad Laboratories, CA, USA) three

times. The blots were further incubated with horseradish peroxidase-conjugated second antibodies at room temperature for 1 hour, then washed with TBS-T three times. The blots were developed with enhanced chemiluminescence (Amersham, Bucks, UK) and then exposed to an iBright 1500 imaging analyzer (Invitrogen, CA, USA).

4 ELISA

To determine the p24 level in the cell-free supernatants, the supernatants were isolated from the culture media at different time points collected from cultures of cells that transiently or stably expressed the ribozymes and were infected with HIV-1. Moreover, the supernatants were filtered through the 0.22 μm polyether sulfone membranes syringe filters (Corning, NY, USA) to diminish the cell debris. The quantification of HIV p24 was performed in captured virus lysates and virus-containing supernatants with the Duo-Set p24 ELISA kit following the manufacturer's (R&D Systems, Minneapolis, MN, USA) instructions. Absorbance readings (450 nm) were performed on a multimode plate reader (Spectra-Max i3, Molecular Devices, Sunnyvale, CA, USA) equipped with Gen 5 software (v. 3.08).

5 RNA Extraction and Quantification

RNA was isolated from iMg (appx. 1.7 x 10^5 cells in total) in 96-well plates in each group with the guidelines described previously. Total RNAs were extracted with Tri-reagent (Molecular Research Center, OH, USA). Prior to reverse transcription, RNA concentrations were measured spectrophotometrically by Nanodrop One (Thermo Scientific™, MA, USA). RNA (1 μg) was subjected to reverse transcriptase PCR using reagents from Pro-mega (Promega, WI, USA). The cDNA sample was then subjected to real-time PCR using iQ SYBR Green Supermix (Bio-Rad Laboratories, CA, USA). All values were normalized to GAPDH mRNA. The sequences of oligonucleotide primers used in this study are listed in Table 1.

Generally, 1 mg RNA was used to produce the cDNA for QPCR with Superscripts III First-Strand Synthesis System (Thermo Scientific™, MA, USA) according to the manufacturer's protocol. And 1.6 μL of cDNA was mixed with 7.6 nuclease-free water, 0.4 μL of 10 mM forward primer, 0.4 μL of 10 mM reverse, primer, and 10 μL of SYBR Green qPCR Master Mix (Thermo Scientific™, MA, USA), qPCR reactions were performed on the StepOnePlus Real-Time PCR System (Applied

Biosystems). Thermocycling conditions were as follows: 95°C for 20 s, 45 cycles of 95°C, for 3 s, and 60°C for 30 s. The difference between the Ct values of the genes of intense rest and the GAPDH gene was calculated for each experimental sample and 2^(- DD Ct) was used to calculate the change in the expression of the genes of interest. It is demonstrated that the efficiencies of the RT-PCR for those genes of interest and the endogenous control were approximately equal.

6 HIV Reverse Transcriptase (RT) Assay

The determination of HIV RT activity was improved in our previous publications and adapted from Dr. Willey's method. In brief, collected culture supernatant (10 µL) was added to a cocktail containing poly (A) (GE Healthcare, Mickleton, NJ), oligo (dT) (USB, Cleveland, OH), MgCl2, and 32P dTTP (PerkinElmer, Boston, MA) and consequently incubated for overnight at 37°C. The cocktail (30 µL) was spotted onto DE81 paper, dried, and washed five times with 2× saline-sodium citrate buffer (Corning, NY, USA) coup once with 95% ethanol. The filter paper was then air-dried or dried in a laboratory oven (Grieve-Hendry, IL, USA). Radioactivity was counted in a liquid scintillation counter (PerkinElmer, Boston, PA).

7 Immunofluorescence

At the outset, iMg were cultured at a density of 5.6×10^4 cells/well in 96-well plates. Subsequently, cells were perfusion-fixed with 4% paraformaldehyde (Sigma Aldrich, MO, USA) for 5 mins coupling with penetration by 1% Triton X-100 (Thermo Scientific™, MA, USA) for 15 mins. Afterward, the plates were blocked with BSA and washed with PBS-T. iMg were ultimately stained with a CD4 and IBA-1 fluorescent for membrane and Hoechst 33342 (Thermo Scientific™, MA, USA) for nuclei and washed three times with 1× PBS. The cells were photographed under a confocal microscope (Nikon A1R, Nikon, Japan).

8 Statistical Analysis

Data were expressed as mean ± standard deviation (mean ± SD) of three individual experiments using iMg from three different donors, donor number are CHOPWT6 P 22,FFP3, CHOPWT 10.2 P40, FFP2, CHOPWT15.2, P30,FFP5. Statistical significance was measured by Student's t-test and One-way ANOVA using GraphPad Prism 11 Statistical Software (GraphPad Software, La

Jolla, USA). *P<0.05, **P<0.01, and ***P<0.001 indicate statistic difference between compared groups.

Table 1 Primers' Pairs

Gene	Orientation	Sequence (5'-3')
GAPDH	Forward	GGTGGTCTCCTCTGACTTCAACA
	Reverse	GTTGCTGTAGCCAAATTCGTTGT
CCR5	Forward	TCTCTTCTGGGCTCCCTACAAC
	Reverse	CCAAGAGTCTCTGTCACCTGCA
CXCR4	Forward	CTCCTCTTTGTCATCACGCTTCC
	Reverse	GGATGAGGACACTGCTGTAGAG
MIP1α	Forward	CTAGAATTCGCGCCATATGGAGCTGACAC
	Reverse	GTCCTCGAGTCAGGCATTTAGTTCCAGCTCAGT
MIP1β	Forward	GCTTCCTCGCAACTTTGTGGTAG
	Reverse	GGTCATACACGTACTCCTGGAC
RANTES	Forward	CCTGCTGCTTTGCCTACATTGC
	Reverse	ACACACTTGGCGGTTCTTTCGG
IFNα	Forward	TTTCTCCTGCCTGAAGAACAG
	Reverse	GCTCATGATTTCTGCTCTGACA
IFNβ	Forward	GCCGCATTGACCATCTATGAGA
	Reverse	GAGATCTTCAGTTTCGGAGGTAAC
IFNλ1	Forward	CTTCCAAGCCCACCCCAACT
	Reverse	GGCCTCCAGGACCTTCAGC
ISG15	Forward	GGCTGGGAGCTGACGGTGAAG
	Reverse	GCTCCGCCCGCCAGGCTCTGT
ISG56	Forward	TTCGGAGAAAGGCATTAGA
	Reverse	TCCAGGGCTTCATTCATAT
VIPERIN	Forward	TGGGTGCTTACACCTGCTG
	Reverse	TGAAGTGATAGTTGACGCTGGT
MX1	Forward	GGCTGTTTACCAGACTCCGACA
	Reverse	CACAAAGCCTGGCAGCTCTCTA
MX2	Forward	AAAAGCAGCCCTGTGAGGCATG
	Reverse	GTGATCTCCAGGCTGATGAGCT

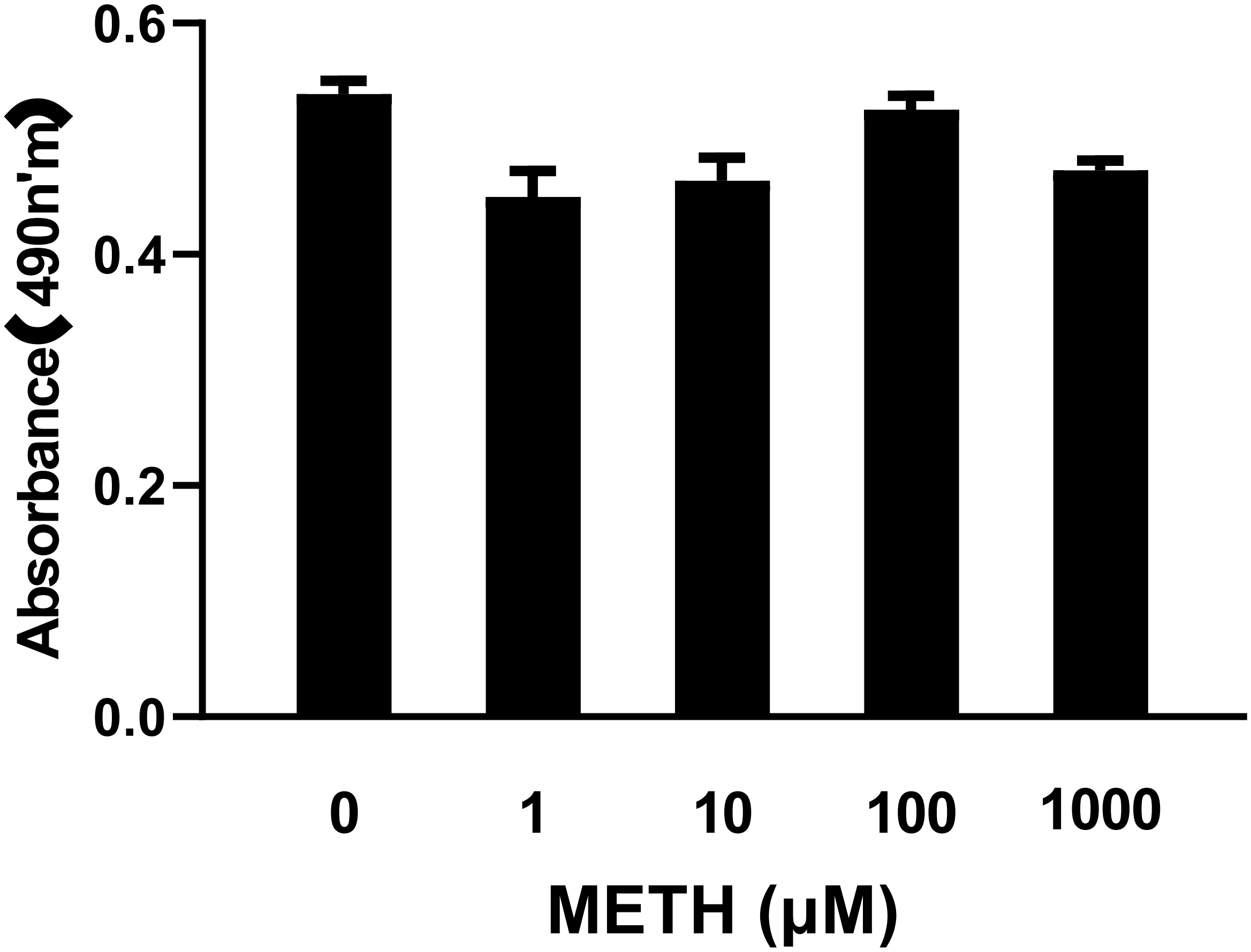

Suppl.3 Cell viability of iMg assessed by MTS assay following METH exposure for 24 hours. No significant cytotoxic effects were observed.
